# Editorial: Migraine and vascular disorders

**DOI:** 10.3389/fpain.2022.964161

**Published:** 2022-07-14

**Authors:** Alessandro Pezzini

**Affiliations:** Department of Clinical and Experimental Sciences, Neurology Clinic, University of Brescia, Brescia, Italy

**Keywords:** migraine, stroke, vascular disease, cerebral vascular accident, migraine with aura

Migraine is a chronic, complex, neurovascular disease, afflicting 10 to 20% of the population, whose typical presentation includes recurrent headache attacks in association with vegetative symptoms and nervous system hypersensitivity (1). Although attacks may be disabling for the patient, the traditional view is that the disease is an essentially benign condition with no consequences for the brain. Notwithstanding, growing evidence indicates that migraine is closely related to stroke occurrence, altered cerebral artery function particularly during acute attacks, and infarct-like abnormalities at brain MRI, which supports the hypothesis of shared biological mechanisms (2). The articles included in this Research Topic provide data to help us better understand the complex relationship between migraine and vascular brain disorders.

## Overview of the articles included in this research topic

Although hormonal status, particularly the estrogen status, is probably the best known among the factors influencing the natural history of migraine (3), there is a paucity of research exploring whether and, if so, how, it might eventually affect cerebrovascular function in migraineurs. In the setting of a case-control study, Dzator et al. tested the hypothesis that premenopausal women with hormonal migraine have impaired cerebrovascular function by transcranial Doppler ultrasound analysis of participants performed when they were free from migraine attacks and non-menstruating. The Authors observed that people suffering hormonal migraine have lower mean blood flow velocity in the left middle cerebral artery both at rest and during cognitive stimulation (neurovascular coupling) compared to healthy controls. This implicates that hormonal migraine might be related to an underlying cerebrovascular dysfunction and, indirectly, supports the hypothesis—hitherto not definitively proven—of a prominent role of impaired cerebrovascular function in the overall population of migraine patients. Whether thyroid hormones may also be somehow implicated in the relationship between migraine and cerebral vascular disease, particularly through the involvement of small vessels, is an intriguing working hypothesis to which the original research article by Guo et al., reported in this Research Topic, provides an indirect cue.

Cardiac abnormalities, particularly patent foramen ovale (PFO) are another factor that has long been debated to explain the link between migraine and cerebral vascular events, again with inconclusive results. A number of observational studies have shown a higher prevalence of PFO among patients with migraine, especially migraine with aura (MA), than among non-migraine subjects (4, 5) and a frequency of MA among PFO-carriers that is almost twice as high as that among non-carriers (6, 7). More recently, MA was associated to the occurrence of ischemic stroke of unknown etiology (cryptogenic ischemic stroke, CIS) though it remains undetermined whether such an association is dependent on the coexistence of PFO (8, 9). Findings reported in this Research Topic by Gollion et al., from a cross-sectional study of 202 patients aged 18 to 54 years, consecutively admitted to a single French academic center because of a first-ever CIS, provide further support to the hypothesis of a triangular, causal relation between PFO, MA and CIS. Though the Authors did not find any significant influence of migraine status on the rate of CIS, MA was associated with PFO showing features (i.e., large shunt and/or co-existent atrial septal aneurysm) that suggest a possibly causal relationship with emboli-related stroke.

Another topic that has been the focus of intense clinical research over the last few years is that of magnetic resonance abnormalities of unclear significance in migraineurs. White matter hyperintesities (WMHs), detected on T2-weighted or fluid-attenuated inversion recovery (FLAIR) images, is the most common of these abnormalities, involving 6–40% of patients with active migraine (2). The differential diagnosis of these covert lesions is not always straightforward. Moreover, the detection of such abnormalities may generate unjustified fears in patients, thus leading to overtesting and, sometimes, overtreatment. In a few cases, WMHs and migraine can be part of the clinical spectrum of an underlying disorder, including small vessel ischemic disease and demyelinating disease. Currently, little information is available on how to distinguish WMHs attributable to migraine from WMHs related to other etiologies. In their original research article, Chong et al. attempted to shed light on this clinical dilemma by analyzing the characteristics of WMHs in a series of 263 patients with migraine. The Authors observed that the majority of WMHs detected in the T2-weighted—FLAIR images of migraineurs were (1) located in lobar regions (especially within the frontal lobe), (2) smaller than 3 mm in diameter, (3) more commonly punctate in shape, (4) non-confluent, and (5) with no corresponding hypointensity on T1-weighted imaging. Furthermore, white matter lesions involving periventricular regions, basal ganglia, brainstem or other structures within the posterior fossa were detected in a small minority of cases. Although many open questions do remain, the Authors concluded that these and other characteristics might help clinicians to differentiate WMHs attributed to migraine from those attributed to other diseases.

## Conclusion

As the articles in this Research Topic demonstrate, there are solid arguments in favor of the hypothesis of a biological link between migraine and cerebral vascular disorders. Identifying the subgroup of migraine patients at increased risk of vascular disease should be the goal of basic and clinical research.

## Author contributions

The author confirms being the sole contributor of this work and has approved it for publication.

## Conflict of interest

The author declares that the research was conducted in the absence of any commercial or financial relationships that could be construed as a potential conflict of interest.

## Publisher's note

All claims expressed in this article are solely those of the authors and do not necessarily represent those of their affiliated organizations, or those of the publisher, the editors and the reviewers. Any product that may be evaluated in this article, or claim that may be made by its manufacturer, is not guaranteed or endorsed by the publisher.
